# Methods for analyzing infant heart rate variability: A preliminary study

**DOI:** 10.1002/bdr2.2177

**Published:** 2023-04-20

**Authors:** Alex Claiborne, Alexandra Williams, Colby Jolly, Christy Isler, Edward Newton, Linda May, Stephanie George

**Affiliations:** 1 Human Performance Laboratory, Department of Kinesiology, East Carolina University, Greenville, North Carolina, USA; 2 Department of Engineering, East Carolina University, Greenville, North Carolina, USA; 3 Obstetrics and Gynecology, East Carolina University, Greenville, North Carolina, USA; 4 Faculty of Family Medicine, East Carolina University, Greenville, North Carolina, USA

**Keywords:** Biocom, ECG, heart rate variability, Hexoskin, Kubios, MATLAB, PPG, R–R interval

## Abstract

Heart rate (HR) and heart rate variability (HRV) reflect autonomic development in infants. To better understand the autonomic response in infants, reliable HRV recordings are vital, yet no protocol exists. The purpose of this paper is to present reliability of a common procedure for analysis from two different file types. In the procedure, continuous electrocardiograph recordings of 5–10 min are obtained at rest in infants at 1 month of age by using a Hexoskin Shirt-Junior’s (Carre Technologies Inc., Montreal, QC, Canada). Electrocardiograph (ECG; .wav) and R–R interval (RRi; .csv) files are extracted. The RRi of the ECG signal is generated by VivoSense (Great Lakes NeuroTechnologies, Independence, OH). Two MATLAB (The MathWorks, Inc., Natick, MA) scripts converted files for analysis with Kubios HRV Premium (Kubios Oy, Kuopio, Finland). A comparison was made between RRi and ECG files for HR and HRV parameters, and then tested with *t* tests and correlations via SPSS. There are significant differences in root mean squared successive differences between recording types, with only HR and low-frequency measures significantly correlated together. Recording with Hexoskin and analysis with MATLAB and Kubios enable infant HRV analysis. Differences in outcomes exist between procedures, and standard methodology for infant HR analysis is needed.

## 1 | INTRODUCTION

Heart rate (HR) and heart rate variability (HRV) are established noninvasive measures of cardiac autonomic function in humans (Carney et al., 1995; Fauchier, Babuty, Cosnay, & Fauchier, 1999; Singh et al., 2018; Tsuji et al., 1996; Vuoti et al., 2021) and could be used as a marker of healthy development in infants (Eiselt et al., 1993; Javorka et al., 2017; Kozar et al., 2018). HRV represents the variation in the duration of the R–R interval (RRi) from successive heartbeats across a cardiogram (e.g., electrocardiogram, ECG) collected for a long (24 hr) or short-term (5–10 min) period (Javorka et al., 2017). Numerous investigations have assessed neonatal HRV as a biomarker of autonomic function (David, Hirsch, Karin, Toledo, & Akselrod, 2007; Javorka et al., 2017) and health and stress in infants (Latremouille, Lam, Shalish, & Sant’Anna, 2021; Longin, Schaible, Lenz, & König, 2005), yet commonization of the methodology is needed to ensure that data are accurately collected and interpreted (Latremouille et al., 2021; Zizzo, Kirkegaard, Uldbjerg, Hansen, & Mølgaard, 2022). Thus, we present accurate outputs using the methodology for infant HRV collection from our group as a means for standardizing future procedures.

Different indices of HRV in the time (RMSSD, root mean square of successive differences; SDNN, standard deviation of the N–N interval) and frequency domains (LF, HF) can be captured from short-term recordings to reflect the balance of different branches of the autonomic nervous system (ANS) in neonates (Chiera et al., 2020; Javorka et al., 2017; Latremouille et al., 2021). The ANS consists of the sympathetic nervous system (SNS), which increases HR in response to stress, and the parasympathetic nervous system (PNS), which is responsible for decreased HR during periods of rest. The assumed dominance of the PNS during rest is reflected by increased time domain HRV, and a high value of high-frequency (HF) power spectral density (PSD), while both the PNS and SNS are reflected in low-frequency (LF) power (Hinde, White, Armstrong, & Altini, 2021; Malik et al., 1996; Shaffer & Venner, 2013).

In adults, reduced HRV is associated with poor cardiovascular function and disease states including diabetes, obesity, and hypertension (Oliveira et al., 2019; Shaffer, Mccraty, Zerr, & Kemp, 2014; Shah et al., 2019). In developing fetuses and infants, HRV is associated with birth status and stress (Lochan Yadav et al., 2017), normal and healthy development and the stress response (Benichou et al., 2018; Chatow, Davidson, Reichman, & Akselrod, 1995; De et al., 2007; Eiselt et al., 1993; Krueger, van Oostrom, & Shuster, 2010; Mazursky, Birkett, Bedell, Ben-Haim, & Segar, 1998; Patural et al., 2004; Patural et al., 2008; van Ravenswaaij-Arts, Hopman, Kollee, Stoelinga, & van Geijn, 1994) and could represent normal development of the autonomic system throughout the late stages of pregnancy and first year of life (Nakamura, Horio, Miyashita, Chiba, & Sato, 2005; Smarius et al., 2018; van Leeuwen, Geue, Lange, Hatzmann, & Grönemeyer, 2003). Furthermore, HRV is considered a reliable biomarker of condition in infants in critical care units (Chiera et al., 2020). Numerous research groups show increased HRV alongside the early development of the cardiac autonomic system (Buyuktiryaki et al., 2018; David et al., 2007; Krueger et al., 2010). Furthermore, HRV has been shown to be an index of attention regulation, orientation in neonates, and early life information processing (Cardoso, Silva, & Guimarães, 2017; Dietz et al., 2016; Zeegers et al., 2018). Others have used HRV to study the effects of maternal exercise during pregnancy, the influences of different delivery modes, the effects of excessive infant crying, the evaluation of prolonged pain in preterm infants, and the influences of parents’ mindfulness on physical emotion regulation of infants. Methodologies for infant HRV recordings used in previous studies are summarized in Table 1. The power bands consist of VLF, LF, and HF. For adults, the accepted ranges of VLF, LF, and HF are 0.0033–0.04, 0.04–0.15, and 0.15–0.4 Hz, respectively (Shaffer & Venner, 2013). Currently, there is no advised range for fetuses or infants (Latremouille et al., 2021).

There is potential for HRV to accurately and non-invasively track infant health. However, some reviews have highlighted differences in methodology between studies and the evidence compiled thus far is of weak quality (Chiera et al., 2020; Latremouille et al., 2021; Moyer, Livingston, Fang, & May, 2015) despite recommendations made from a Clinical Task Force (Table 2). The different methods for recording and HRV analysis have made interpretation of HRV in infants difficult (May, Glaros, Yeh, Clapp, & Gustafson, 2010). Variations in recording periods and digital processing introduce unwanted variance in the data. The current study probed variances in two different analyses used to measure and calculate infant HRV and present a standard methodology for future investigations.

## 2 | MATERIALS AND METHODS

### 2.1 | Study design

This study is an analysis of infant HRV recordings. The overall study was focused on determining the influence of exercise during pregnancy on infant health outcomes (Bartels, Neumamm, Peçanha, & Carvalho, 2017; May et al., 2014; Wang & Huang, 2012). The study population was comprised of 1-month-old infants born to women enrolled in a partially blinded, prospective two-arm randomized controlled trial. Written maternal informed consent was obtained from each participant prior to study enrollment. This study was approved by the East Carolina University Institutional Review Board.

### 2.2 | HRV analysis

**FIGURE 1:**
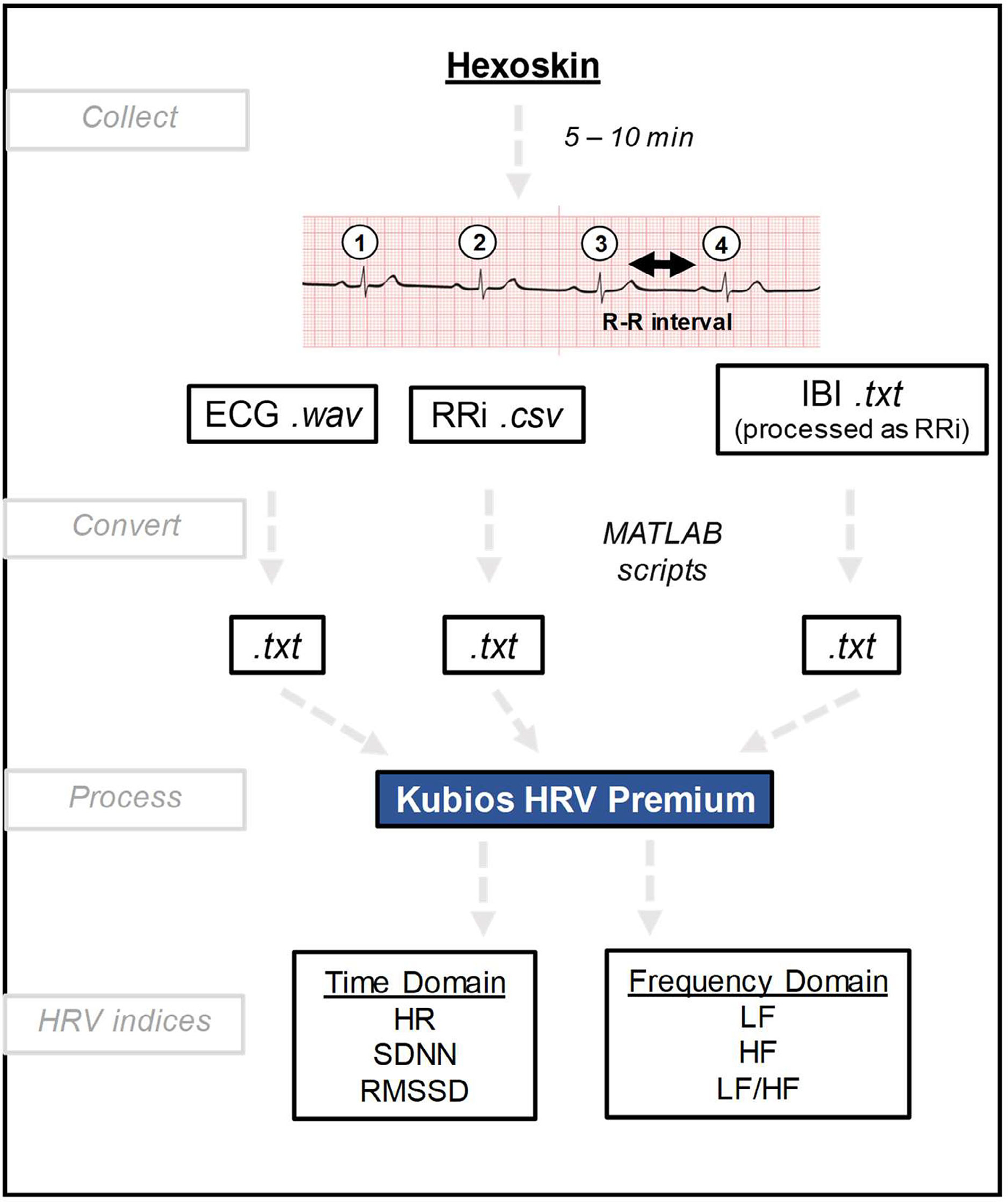
Schematic of HRV collection and processing. 5–10 min readings of ECG (electrocardiograph) are converted to different file types for ECG (.wav), RRi (.csv), and IBI (.txt) analysis. All file types are converted to .txt and read in Kubios HRV Premium for extraction of HRV indices. HRV, heart rate variability.

In accordance with Task Force guidelines (Shaffer & Venner, 2013), continuous ECG signals of 5–10 min were recorded on infants at 1 month of age by using a Hexoskin Shirt (Carre Technologies Inc., Montreal, QC, Canada). HR and HRV were recorded (Figure 1) when the infants were in a quiet but alert state in the supine position. From the Hexoskin files, both the ECG (.wav) and RRi (.csv) files were generated automatically by the VivoSense Software (Great Lakes NeuroTechnologies, Independence, OH). Step-by-step protocol can be found in supplementary file “S1_Protocol.”

Two MATLAB scripts (The MathWorks, Inc., Natick, MA) were used to prepare the files to be processed through Kubios (Figure 1). The first code extrapolated the RRi files between 4 and 5 min, added 10 s to the beginning of each file, and converted the RRi files from .csv to .txt. The second code converted the ECG files from .wav to .txt. It has been determined that the frequency domain parameters cannot be calculated directly from the RRi due to correction procedures (Mali, Zulj, Magjarevic, Miklavcic, & Jarm, 2014; Pichon, Roulaud, Antoine-Jonville, de Bisschop, & Denjean, 2006; Suga et al., 2019; Yiallourou et al., 2012). An even sampled signal is needed, which is most commonly done through a 4 Hz cubic spline interpolation. The signal is converted into the frequency domain by using the Fast Fourier transform (FFT; Mali et al., 2014). RMSSD was calculated as: $\text{RMSSD}\;=\;\sqrt{\frac{1}{N-1}}\sum_{j=1}^{N-1}{\left(RR_{i+1}-RR_{i}\right)}^{2}$. The frequency domain parameters are based on the estimated PSD of the normal-to-normal interval. The PSD ranges used for LF and HF were: 0–0.04 Hz, 0.04–0.2 Hz, and 0.2–1.5 Hz for VLF, LF, and HF, respectively.

Since 5 min of data is recommended to calculate time and frequency parameters of HRV, ECG, and RRi files less than 4 min were discarded. Ten infant recordings were included with both ECG and RRi files converted to .txt files. The software used to analyze the HRV parameters of RRi and ECG signals was Kubios HRV Premium (Kubios Oy, Kuopio, Finland; Figure 1).

### 2.3 | Statistical analyses

Two-sided *t* tests were used to test for differences in RRi and ECG signals to analyze infant heart rate (HR), RMSSD, SDNN, LF, HF, and LF/HF ratio. Correlations were used to look for relationships in infant HR and HRV measures between the two methods. Statistical analyses were performed using SPSS (Statistical Package for Social Sciences) v.25. Statistical significance was determined a priori at *p* < .05. For Bland–Altman plot analysis, the average of ECG and RRi variables for each subject were calculated, then divided by 2, and plotted against the mean difference. Lines were applied for the 95% limits of agreement, and standard deviation was calculated for sample versus population. Finally, a regression analysis of the points on the plot was performed to assess proportional bias.

## 3 | RESULTS

**FIGURE 2:**
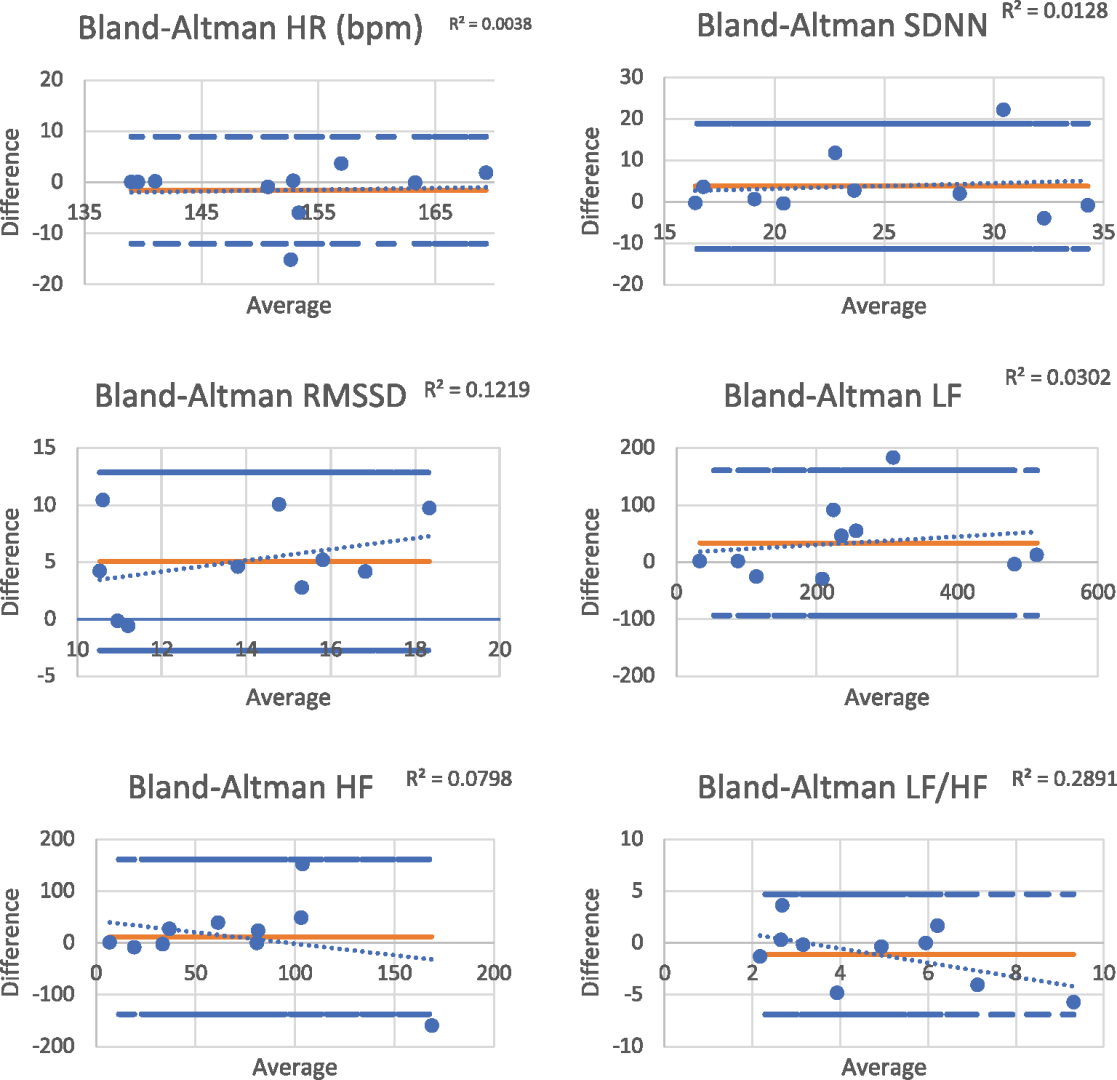
Bland–Altman plots for HRV reliability between measures. HRV, heart rate variability.

This procedure (S1_Protocol) successfully produced infant HRV outputs comparable to other work from our group (Bartels et al., 2017), using Hexoskin for recording, instead of magnetocardiogram. The HR and HRV data between the ECG and RRi files are similar, except for RMSSD (Table 3). Additionally, Bland–Altman plots lend that the two methods are in agreement for HR, SDNN, RMSSD, LF, and HF (Figure 2). A significant fixed bias was only observed for RMSSD (*p* < .05). Low *R*^2^ from the individual data points on the Bland–Altman plot indicates a lack of proportional bias between methods. However, only HR and LF have significant correlations between the ECG and RRi files (Table 4), indicating further processing occurs with the RRi files by VivoSense. A subset of the infants included in the study also had an ECG completed. Correlation of 1.00 demonstrated a perfect correlation of the ECG with both the Hexoskin ECG and RRi infant recording files.

## 4 | DISCUSSION

The purpose of this paper is to present different procedures used to calculate infant HRV. The main findings of this analysis are (a) HR and LF are similar regardless of the processing method, and (b) RMSSD is significantly different between the processing methods. The authors demonstrate the need for a common procedure of analyzing raw cardiographs for time and frequency parameters of HRV. It is prudent that unwanted variance from inconsistent file correction and processing methods be minimized.

Table 3 displays HRV indices between ECG and RRi files, which are consistent with other reports of infant HRV in the first month of life (Bartels et al., 2017; Longin et al., 2005; Pados et al., 2017; Patural et al., 2008). However, the current study shows the RMSSD measure to be more sensitive to variations in methodology, namely file type. Thus, researchers and clinicians should be cautious when comparing time domain HRV across different methods. Noticeable, but statistically insignificant clinical differences were noticed in the other time domain variables, while frequency domain HRV indices seem more robust. The authors believe the root of this problem to be a discrepancy between the processing of the ECG file into an RRi file so that it may be further processed by our MATLAB scripts, and generated into a readable file for final analysis in Kubios.

However, on examination of Table 4, it appears that across all HRV indices included in the current study, only HR and LF were significantly correlated, that is, showed similar trends when compared on the same infant’s recording. This interesting finding suggests that HR and LF are more reproducible between the two methods for processing in the current study. While the underlying reason for this occurrence was not tested in the current study, HR and LF appear more reliable for cross-comparison between studies using different methods. Both time (SDNN and RMSSD) and frequency domain (LF, HF, and LF/HF) parameters were not correlated to each other from the ECG and RRi files, indicating that the recordings are processed differently. When RRi files are uploaded into Kubios for determination of HRV, artifacts cannot be manually corrected; only automatic correction methods can be used, which are advised against by the Task Force due to common error (Shaffer & Venner, 2013). On the contrary, artifacts can be manually corrected when ECG files are uploaded. In essence, using the ECG signal gives the user more control to edit the signal if they observe artifacts or missed identification of R wave peaks. As validation that the ECG file types still provide accurate HR data, Table 4 illustrates the correlation in HR values with the RRi and ECG signals.

Once ECG files are processed for HRV, additional unwanted variance is seen in the interpretation of PSD modeling. For frequency domain HRV, the power spectral ranges consist of VLF, LF, and HF (Mali et al., 2014; Pichon et al., 2006; Suga et al., 2019; Yiallourou et al., 2012). For adults, the accepted ranges of VLF, LF, and HF are 0.0033–0.04, 0.04–0.15, and 0.15–0.4 Hz, respectively (Mali et al., 2014; Pichon et al., 2006; Suga et al., 2019; Yiallourou et al., 2012). Currently, there is no advised standard range for fetuses or infants (Bartels et al., 2017; Kozar et al., 2018; Latremouille et al., 2021; Wang & Huang, 2012) though HRV is already implemented in infant clinical practice (Chiera et al., 2020). May et al. (Bartels et al., 2017; Wang & Huang, 2012) indicate the frequency ranges were based on the work of David and van Leeuwen (Buyuktiryaki et al., 2018; David et al., 2007), while Kozar et al. (Kozar et al., 2018) base their frequency ranges on the manuscript by Javorka et al. (Javorka et al., 2017). These and other articles acknowledge that standardization is needed for proper interpretation (Latremouille et al., 2021). The ranges used by May et al. (Bartels et al., 2017) were 0–0.04, 0.04–0.2, and 0.2–1.5 Hz for VLF, LF, and HF, respectively. For Kozar et al. (Kozar et al., 2018), the LF range was 0.04–0.15 Hz, and the HF range was 0.15–1.5 Hz; Smarius et al. (Zeegers et al., 2018) did not indicate what ranges were used for LF and HF; Suga et al. (Shepherd et al., 2021) used .04–.24 for LF and .24–1.04 for HF. Kozar et al. (Kozar et al., 2018) use the same LF range as adults, while May et al. use a slightly longer LF range. The LF ranges in these studies differ by 0.05 Hz, and the studies use a similar upper limit of 1.5 Hz for HF. Most groups extend the HF band to 1.33 Hz (Pados et al., 2017) or 1.5 Hz (Bartels et al., 2017; Javorka et al., 2017; Kozar et al., 2018) to account for increased infant respiratory rate. The HF upper limit is over three times larger than the HF upper limit seen for adults.

Of the investigations included in Table 1, there are many variations in the use of tracing hardware, sampling frequency, recording duration, analysis software, and PSD parameters. In line with the recommendations of the Task Force, ECG tracings should be sampled at a rate of >200 Hz, and epochs not less than 5 min duration should be analyzed for HRV (Shaffer & Venner, 2013). Based on differences in hardware reliability, and further filtration and processing in the software, as shown in VivoSense, the authors recommend the use of programs such as Kubios which have been shown to not further process ECG or RRi files before displaying HRV metrics. A limitation of the study is that the findings were tested with respect to the VivoSense package, but other software was not tested and therefore could show different conclusions. Finally, future studies should focus on standardization of the HF component of frequency domain HRV to a specific range to allow confident comparison between studies from different groups.

## Supplementary Material

Supplemental Info

## Figures and Tables

**TABLE 1:** Variations in methodology among infant HRV literature

Study	Infant age	Cardiograph tracings	Sampling frequency	HRV analysis duration	Analysis + correction	HRV parameters		Frequency (Hz)
						Time	Frequency	
May et al.; (May et al., 2010; May, Scholtz, Suminski, & Gustafson, 2014)	1 mo.	Magnetocardiogram (MCG)	300 Hz	18 min	EEGLAB + MATLAB; Automatic + manual	SDNN RMSSD	VLF, LF; HF, LF/HF	LF 0.04–0.2; HF 0.2–1.5
Kozar et al. (Kozar et al., 2018)	1–4 d.	Electrocardiograph (ECG)	1,000 Hz	300 RRi	Automatic + manual	Rri MSSD;	LF, HF	LF 0.04–0.15; HF 0.15–1.5
Smarius et al. (Smarius et al., 2018)	5 yr.	Electrocardiograph (ECG) or impedance cardiograms (ICG)	-	4 min	VU-AMS; manual	-	LF, HF	-
Zeegers et al. (Zeegers et al., 2018)	4 mos.; 12 mos.	Electrocardiograph (ECG)	200 Hz	2 min	Vsrrp98; automatic	RMSSD; SDNN	-	-
Shepherd et al. (Shepherd et al., 2021)	1–4 wks.	Electrocardiograph (ECG)	400 Hz	1–2 min	MATLAB; manual	-	LF, HF; TP, LF/HF	LF 0.04–0.15; HF 0.4–1.5
Yiallourou et al. (Yiallourou, Sands, Walker, & Horne, 2012)	3, 10, 22 wks.	Electrocardiograph (ECG)	400 Hz	1–2 min	MATLAB; automatic	-	LF, HF; TP, LF/HF	LF 0.04–0.15; HF: respir.
Suga et al. (Suga, Uraguchi, Tange, Ishikawa, & Ohira, 2019)	3–8 mos.	Electrocardiograph (ECG)	-	5 min	RRi analyzer 2; UNION TOOL Co., Japan	-	LF, HF	LF mom 0.04–0.15; Infant 0.04–0.24; HF mom 0.15–0.4; Infant 0.24–1.04;
Pados et al. (Pados, Thoyre, Knafl, & Nix, 2017)	<8 mos.	Electrocardiograph (ECG)	1,000 Hz	2 min	MindWare HRV; Automatic + manual	-	HF	HF 0.3–1.33

*Note:* Representative sample of peer-reviewed reports of HRV in infants varying ages 1 day to 5 years.

Abbreviations: HF, high frequency; HRV, heart rate variability; Hz, hertz; LF, low frequency; min, minute; RMSSD, root mean squared standard deviation; RRi, R-R interval; SDNN, standard deviation of N-N interval; TP, total power; VLF, very low frequency.

**TABLE 2:** Task Force HRV analysis recommendations

Epochs of 5 min for power spectral analysis
Use of a sampling rate at least 250 Hz
Sampling while heart rate is steady
Use of manual identification and filtering of artifact
Time domain analysis for long-term recordings
Standardize body position during sampling
PSD ranges: 0.004–0.04–0.15–0.4 Hz (adults) Not specified (infants)

*Note:* Existing advice for analysis of HRV in adults and infants, summarized from the Task Force of the European Society of Cardiology and North American Society of Pacing Electrophysiology.

Abbreviations: HRV, heart rate variability; PSD, power spectral density.

**TABLE 3:** HR and HRV data between ECG and RRi files

HRV measure	ECG (*n* = 10)	RRi (*n* = 10)	*p* Value
HR (bpm)	151.1 ± 10.5	152.7 ± 10.2	.74
SDNN (ms)	26.4 ± 7.9	22.6 ± 7.2	.28
RMSSD (ms)	16.4 ± 4.0	11.3 ± 2.8	**.004**
LF (ms^2^)	263.2 ± 165.2	229.3 ± 154.2	.64
HF (ms^2^)	75.5 ± 52.7	63.5 ± 69.8	.67
LF/HF	4.3 ± 2.0	5.4 ± 3.4	.38

*Note:* Data are presented as mean ± SD. *p* Value calculated from *t* tests. Abbreviations: bpm, beats per minute; ECG, electrocardiograph; HF, high frequency; HR, heart rate; HRV, HRV, heart rate variability; LF, low frequency; ms, milliseconds; RMSSD, root mean squared standard deviation; RRi, R-R interval (file type); SDNN, standard deviation of N-N interval. Bold indicates significant values.

**TABLE 4:** Correlation in HRV between ECG and RRi

HRV measure	Correlation	*p* Value
HR (bpm)	.867	**.001**
SDNN (ms)	.483	.16
RMSSD (ms)	.364	.30
LF (ms^2^)	.920	**<.001**
HF (ms^2^)	.250	.49
LF/HF	.482	.16

*Note*: Data are presented as correlation coefficient.

Abbreviations: bpm, beats per minute; ECG, electrocardiograph; HF, high frequency; HR, heart rate; HRV, HRV, heart rate variability; LF, low frequency; ms, milliseconds; RMSSD, root mean squared standard deviation; RRi, R-R interval (file type); SDNN, standard deviation of N-N interval. Bold indicates significant values.

## Data Availability

Deidentified data may be made available upon request to Dr. Linda May, lead investigator.
