# Supplementary material for: Methods for analyzing infant heart rate variability: A preliminary study
Source: Birth Defects Res. Author manuscript; Available in PMC 2024 Jul 5. (PMC11226182; doi:10.1002/bdr2.2177)
Supplement: Supplemental Info [file NIHMS2002281-supplement-Supplemental_Info.pdf]

S1 Protocol for Infant Heart Rate Variability Analysis

1) Ensure Hexoskin device is fully charged before recording. A full charge takes ~ 2 hours.
2) Either in your web browser, or in the Hexoskin application, create an account so that you may upload the recording.
3) Register your device's serial number and assign to your account.
4) Disconnect the Hexoskin device from your computer, and ensure the device is not currently recording (2nd orange LED is extinguished).
    a. If using the application, pair the Hexoskin to your phone or tablet via Bluetooth - OR- use the Hexoskin mobile app to pair.
5) To dress the shirt on your participant:
    a. Fold up the lower part of the shirt.
    b. Put one arm through the first arm opening.
    c. Carefully move the head opening over your participant's head.
    d. Place the other arm opening over the other arm.
    e. Fold down the lower part of the shirt.
    f. Gently wet the 3 gray electrodes with water or gel.
6) Plug your Hexoskin device into the shirt, and place in shirt pocket. Doing so will automatically turn the device on and begin recording.
7) Obtain 5–10-minute electrocardiograph recordings on infants using Hexoskin Shirt.
    a. Ensure infants are in a quiet but alert state.
    b. Measurements should be made in the supine position.
    c. To stop recording, unplug the Hexoskin device.
8) Sign into your Hexoskin account in HxServices, then plug Hexoskin device into your computer to begin upload.
    a. Select your device, then click "sync".
    b. Data will be available on your Hexoskin dashboard in the application, or online at my.hexoskin.com.
9) Extract ECG (.wav) and R-R interval (.csv) files.
    a. Using Vivosense software, both ECG (.wav) and RRi (.csv) files will be generated upon uploading the Hexoskin recording.
    b. Generate an RRi file from the ECG .wav file in Vivosense.
10) Convert both RRi files to .txt using MATLAB scripts.

    The first code:
    1) extrapolates the RRi files between 4-5 minutes
    2) adds 10 seconds to the beginning of each file
    3) converts the RRi files from .csv to .txt

    The second code:
    1) converts the ECG files from .wav to .txt
11) Analyze the .txt files separately using Kubios HRV Premium, using Fast Fourier transform (FFT) to estimate power spectral density parameters.
12) Remove files of recordings less than 4 minutes from analysis, per recommendations.
